# Oral Microbiota-Driven Cell Migration in Carcinogenesis and Metastasis

**DOI:** 10.3389/fcimb.2022.864479

**Published:** 2022-04-29

**Authors:** Huimin Bai, Jing Yang, Shu Meng, Chengcheng Liu

**Affiliations:** ^1^ State Key Laboratory of Oral Diseases, National Clinical Research Center for Oral Diseases, Department of Periodontics, West China School & Hospital of Stomatology, Sichuan University, Chengdu, China; ^2^ State Key Laboratory of Oral Diseases, National Clinical Research Center for Oral Diseases, Department of Cariology and Endodontics, West China School & Hospital of Stomatology, Sichuan University, Chengdu, China

**Keywords:** oral microbiota, cell migration, EMT, carcinogenesis, metastasis

## Abstract

The oral cavity harbors approximately 1,000 microbial species, and both pathogenic and commensal strains are involved in the development of carcinogenesis by stimulating chronic inflammation, affecting cell proliferation, and inhibiting cell apoptosis. Moreover, some substances produced by oral bacteria can also act in a carcinogenic manner. The link between oral microbiota and chronic inflammation as well as cell proliferation has been well established. Recently, increasing evidence has indicated the association of the oral microbiota with cell migration, which is crucial in regulating devastating diseases such as cancer. For instance, increased cell migration induced the spread of highly malignant cancer cells. Due to advanced technologies, the mechanistic understanding of cell migration in carcinogenesis and cancer metastasis is undergoing rapid progress. Thus, this review addressed the complexities of cell migration in carcinogenesis and cancer metastasis. We also integrate recent findings on the molecular mechanisms by which the oral microbiota regulates cell migration, with emphasis on the effect of the oral microbiota on adhesion, polarization, and guidance. Finally, we also highlight critical techniques, such as intravital microscopy and superresolution microscopy, for studies in this field.

## Introduction

An eclectic and diverse assemblage of microbiota inhabits different sites within the oral cavity, such as teeth, saliva, and oral mucosal surfaces (Lamont et al., 2018). Distinct microbial communities accumulate in these sites through successive colonization events. Since van Leeuwenhoek made the first observation of oral bacteria in dental plaques using primitive microscopes in 1683, our knowledge of oral microbiology has burgeoned. Investigations of the oral microbiota in health and diseases are currently undergoing rapid progress due to the development of techniques (Willis and Gabaldon, 2020). The oral microbial community is a complex and dynamic entity, and shifts in these communities contribute to both oral diseases (e.g., dental caries, periodontitis) and systemic diseases (e.g., diabetes mellitus, cardiovascular disease) (Kleinstein et al., 2020; Hajishengallis and Lamont, 2021). Interestingly, the carcinogenic effect of the microbiome in various organs has been revealed by studies in germ-free animals (Scott et al., 2019). Recently, the microbiome has been considered an influential environmental factor modulating carcinogenesis (Scott et al., 2019; Irfan et al., 2020). *Helicobacter pylori* is the most known bacterial carcinogen (Alipour, 2021). There is also increasing evidence indicating a correlation of some specific species with cancer, including *Porphyromonas gingivalis*, *Treponema denticola*, *Fusobacterium* sp., *Streptococcus* sp., *Peptostreptococcus* sp., *Prevotella* sp., and *Capnocytophaga* (Fitzsimonds et al., 2020). In some cases, a dysbiotic community results from a shift in microbial composition rather than a specific organism associated with the carcinogenic process (Irfan et al., 2020). The current strategies to study the role of the oral microbiome in cancer have predominantly focused on detecting microbial communities present or populational shifts in specific samples and investigating host responses to specific microbial challenges, such as immunological responses, inflammatory responses, and cell proliferation. The potentially oncogenic oral bacteria and possible mechanisms of their action on the carcinogenesis of cells have been systematically reviewed (Whitmore and Lamont, 2014; Fitzsimonds et al., 2020; Irfan et al., 2020; Lamont et al., 2022). In summary, oral microbiota participate in cancer mainly *via* the following mechanisms: (1) stimulating chronic inflammation; (2) inducing mutagenesis, oncogene activation, and angiogenesis; (3) facilitating cell proliferation and inhibiting cellular apoptosis; and (4) producing carcinogens.

In cancer biology, the microbiome is considered an influential environmental factor modulating the carcinogenic process (Scott et al., 2019). Cell migration is an essential physiological process for the immune response, wound repair, and tissue regeneration, while abnormal cell migration is found in devastating diseases such as tumor formation and metastasis. An increasing number of studies have shown that cell migration can be induced by oral bacteria, such as *P. gingivalis* and *Fusobacterium nucleatum*. The latest study found that the tyrosine phosphatase (Ltp1) of *P. gingivalis* is secreted and facilitates *P. gingivalis*-induced proliferation, migration, and the epithelial to mesenchymal transition (EMT) of gingival epithelial cells by targeting the regulator of growth and cell cycle (RGCC). Three distinct activities occurring either simultaneously or independently are involved in cell migration: protrusion, attachment, and traction. There is a complex and discrepant scenario of these activities depending on conditions and migration types. Taking epithelial cells as an example, slow-moving epithelial cells are characterized by slipping of adhesion and retrograde actin flow, while fast-moving epithelial cells have more gripping adhesions and rapid protrusion (Jurado et al., 2005). Single-cell migration and collective cell migration are two main types of cell migration. Studies have demonstrated that epithelial cells either migrate collectively or undergo EMT, thus migrating as single mesenchymal cells (Lu and Lu, 2021). Migration and EMT are highly compatible and facilitate each other involved in development, wound healing, and cancer metastasis (Son and Moon, 2010; Stone et al., 2016). For example, cell migration induced the spread of highly malignant cancer cells (Mosier et al., 2021). Moreover, carcinoma cells migrate into adjacent tissues and invade the lymphatic system and blood vessels and then seeds in distant organs (Christiansen and Rajasekaran, 2006).

Therefore, cell migration is a potential linkage between oral microbiota and carcinogenesis. Recently, investigations in this field have attracted more attention. However, the mechanisms underlying how the oral microbiota participates in regulating cell migration remain to be elucidated. To contribute to the understanding of this issue, this review summarizes the role of cell migration in carcinogenesis and cancer metastasis and focuses on the mechanisms employed by oral microbiota for regulating cell migration. Finally, we introduced critical techniques in the field of cell migration investigation to arouse exciting science over the next decade.

## Cell Migration

### Cell Migration and EMT

EMT is a cellular biological process involved in tumor cell invasion and metastasis, which was first discovered by Elizabeth Hay during gastrulation in vertebrate embryos in 1982 (Greenburg and Hay, 1982). EMT has been traditionally defined as a process by which epithelial cells lose their vestiges of epithelial origin (e.g., cell–cell adhesion and cell polarity) while migrating and invading into mesenchymal stem cells (Stemmler et al., 2019). Research findings in recent years have found that EMT is not a binary process; there is the hybrid E/M phenotype of cells that originate from epithelial cells in the progression of cancer, known as partial, incomplete, or hybrid EMT (Bakir et al., 2020). The hybrid EMT has been demonstrated to be involved in various human primary cancers (e.g., head and neck cancer) and carcinosarcomas (e.g, esophageal carcinosarcomas) (Pastushenko and Blanpain, 2019). Theoretical and experimental efforts have provided crucial insights into the mechanisms of EMT and the coupling between EMT and other biological processes, such as cell migration, the cell cycle and apoptosis (Lovisa et al., 2015; Aiello et al., 2018; Li et al., 2019; Prakash et al., 2019). The relationship between EMT and cell migration has attracted increasing interest (Aiello et al., 2018; Li et al., 2019). In general, EMT is an important biological process for epithelial-derived cells to acquire migration and invasion during cancer development, especially during metastasis. There is mounting evidence that cancer cells exploit EMT to increase their migratory and invasive ability during the initial stage of the metastatic cascade. Furthermore, the correlation between EMT and migration is type-dependent. A highly specialized epithelial cell is relatively fixed and possesses a low migration ability due to cell–cell adhesions and the apico-basal surface. Therefore, achieving complete mesenchymal transformation is a prerequisite for the migration of a single epithelial cell. During EMT, epithelial cells gain features such as cell elongation, motility, and invasion, coordinated by reorganization of the actin cytoskeleton. In turn, cell migration occurs, which gives rise to single tumor cells capable of crossing basement membranes and invading blood vessels. This process has been utilized by many breast and colorectal cancer cell lines (Aiello et al., 2018). However, the invasion of cancer cells is usually visualized as the migration of groups of cells. The hybrid state of EMT has been associated with increased invasion and collective cell migration, in which cells retain cell–cell adhesion with each other and possess mesenchymal features, such as increased motility and loss of apical–basal polarity. For instance, Campbell *et al.* demonstrated that the hybrid EMT driven by *Snail* induced collective cell migration and seeded polyclonal metastases in *Drosophila* intestinal tumors (Campbell et al., 2019). Overall, cells that undergo EMT obtain increased migratory ability. Key pathways and cellular events that participate in controlling EMT are also considered to be important factors that regulate cell migration.

Three mechanisms of action have been suggested regarding EMT. The first is deconstructing cell junctions and polarity. The typical characteristics of polarized epithelial cells are tight junctions, adhesive junctions, desmosomes, and gap junctions. Over the years, numerous studies have elucidated that the junction complex plays a vital role in EMT as a medium for polarizing cell–cell contact as well as an anchor point for the actin cytoskeleton. Actin-binding proteins (ABPs) and Rho family GTPases participate in regulating the actin cytoskeleton by controlling the polymerization and disintegration of actin filaments (Tokuraku et al., 2020). Adhesive junctions can anchor capillary basal microtubule arrays and participate in collective cell migration *via* the interaction between E-cadherin and discoidin domain receptor 1 (DDR1) (Gadiya and Chakraborty, 2018). Microtubules and intermediate filaments are also critical for EMT by influencing cell motility, cell shape, intracellular trafficking, and forces to support protrusion (Datta et al., 2021). The second mechanism attributed to EMT is cytoskeletal changes and motility. Active remodeling of the cytoskeleton is crucial for the transformation of cells into a more motile phenotype, thereby promoting EMT (Amack, 2021; Leggett et al., 2021). In the third mechanism, mast regulators regulating gene expression that contribute to the repression of epithelial phenotype and activation of the mesenchymal phenotype drive EMT, including Snail, Slug, Twist1, Zeb1 and Zeb2 (Stemmler et al., 2019).

### Cell Migration in Carcinogenesis

Since Pott noticed the association between exposure to soot in chimneys and scrotal cancer in 1775, which is considered the first research describing the cause of cancer, an explosion of research evidence on the etiology of cancer has occurred rapidly. The mechanism of carcinogenesis is a complicated process involving the regulation of various levels and pathways. Exogenous substances (e.g., chemicals) or endogenous signals (e.g., reactive intermediates generated from cellular pathways) can lead to mutations in proto-oncogenes or tumor suppressors (Patterson et al., 2018). The biological characteristics, pathways or genetic alterations during cell migration may also be related to cancer. For instance, the Abl family of tyrosine kinases can modulate actin cytoskeleton arrangement through activation of cell-surface receptors that are critical for cell motility and migration (Bradley and Koleske, 2009). They also play an important role in the progression of leukemia and solid tumors. ABL1 has been identified as an oncogene in leukemia. The upregulation and activation of ABL can also be observed in solid tumors (Greuber et al., 2013).

It has been gradually noticed that actin also exists in the nucleus (Rando et al., 2002). Actin in cytoplasm is the main provider for driving force at the front edge and responsible for the contraction of the cell body at the rear edge. Recent studies have found that increasing the level of nuclear actin monomer inhibited cell migration by regulating serum response factor (SRF) and TEA Domain (TEAD) transcription factors expression (Mcneill et al., 2020). Cell migration can be accelerated when the polymerization of nuclear membrane and actin filament is prevented (Fracchia et al., 2020). ABPs combine with actin to make it pass through the nucleus. Actin cytoskeleton dynamics are the basis of cell migration. The polymerization and degradation of actin filaments influenced by ABPs is an essential step of cell migration. ABP can regulate cell activity mediated by actin elaborately (Tokuraku et al., 2020). Additionally, ABPs induce the production of invasive structures such as filopodia (Wang et al., 2017). Meanwhile, actin and ABPs are involved in the process of carcinogenesis. Nuclear actin, as an important part of the chromatin complex, may affect DNA transcription and repair. The levels of actin and ABPs may be connected to chromatin remodeling and upregulation of oncogenes (Izdebska et al., 2020). Different kinds of ABPs in the nucleus (i.e., α-actinin-4, nerspin and cofilin) are associated with the expression of genes responsible for tumorigenesis and influence tumorigenic phenotypes (An et al., 2016; Sur-Erdem et al., 2020).

During cell migration, it is necessary for migrating cells to reduce cell rigidity and pass through narrow intercellular spaces. Genomic changes, including temporary nuclear envelope rupture, DNA damage and genomic rearrangements, can be observed in the process of immune and cancer cell migration (Denais et al., 2016; Raab et al., 2016). DNA damage is an important first step in carcinogenesis. When the replication of damaged DNA takes place before DNA repair tools come into play or the damage process occurs at a high frequency, nuclear deformation-associated genomic instability may lead to various cellular responses, ultimately leading to cancer (Basu, 2018). In the case of other cell migration, it is still necessary to discuss whether DNA will be damaged mechanically and then cause carcinogenesis.

### Cancer Cell Migration in Metastasis

Cancer cell migration is a critical parameter in metastatic dissemination, which allows the cells to detach from the primary tumor, migrate through the extracellular matrix (ECM), enter the lymphatic vessels or the bloodstream, spread within the tissues and then undergo metastatic growth in distant organs. The same principles of cell migration were employed by cancer cells and nonneoplastic cells (e.g., keratinocytes, fibroblasts) as mentioned above. Histopathological evidence has indicated that cancer cells spread within tissues in diverse patterns. They can disseminate using amoeboid- or mesenchymal-type cell migration or collective migration, which expand into solid cell strands, sheets, files, or clusters. Moreover, multiple forms usually exist simultaneously. Amoeboid-like cells often migrate alone or in streams. Mesenchymal-type cells tend to switch between various modes, including single-cell, in streams and collective migration (Clark and Vignjevic, 2015). For instance, oral squamous cell carcinoma (OSCC) exhibits predominantly collective cell migration when explanted *in vitro*. However, most solid stromal tumors disseminate *via* individual cells. Overall, these patterns are regulated by the molecular repertoire of cancer cells, which mainly includes integrins, matrix-degrading enzymes, cell–cell adhesion molecules and cell–cell communication. Increased contractility mediated by the Rho pathway in cancer cells migrating individually facilitates amoeboid-like migration (Sahai and Marshall, 2003). Under most conditions, the lower the differentiation stage is, the more likely the cancer cells are to spread through a single cell.

In collective migration, cell clusters retain intercellular connections and combine with ECM through integrins, cadherin, gap junctions, etc. (Shih and Yamada, 2012; Yang et al., 2019). Partial retention of epithelial characteristics and partial EMT allow cells to migrate as clusters (Shih and Yamada, 2012; Cheung and Ewald, 2016; Liao et al., 2021). Cells in migrating clusters are usually divided into two groups: leader cells and follower cells. Leader cells sense the cancer microenvironment and create a low-resistance migration path (Vilchez Mercedes et al., 2021). The traction generated by leader cells can be transmitted along with cell–cell junctions to enable the migration of follower cells (Bazellières et al., 2015). Bronsert found that cells at the metastasis front rarely expressed mesenchymal morphology or EMT markers and speculated that single-cell migration is rare or even absent in most epithelial tumors (Bronsert et al., 2014). Collective cell migration may play a key role in the process of cancer cell metastasis. Moreover, cancer seeding by collective migration has a worse clinical prognosis because of its greater metastatic and proliferative potential (Hou et al., 2012; Aceto et al., 2014).When tumor cells need to pass through the narrow channel of extracellular space in dense three-dimensional matrices, they can establish the polarized distribution of Na^+^/H^+^ pump and aquaporin, thus resulting in the inflow of water at the front of cells and the outflow of water at the back of cells, resulting in net cell displacement (Stroka et al., 2014). The method that adjusts the volume through water infiltration provides another possible cell migration mechanism that does not require actin polymerization.

## Oral Microbiota and Cell Migration

### Oral Bacteria Regulating Cell Migration

The oral cavity contains one of the greatest microbiological reservoirs in the human body. The dynamic and finely balanced relationship between the oral microbiome and the host is of great importance to human health (Hajishengallis and Lamont, 2021). Bacteria are the largest contributor to the oral resident microbiota, 94% of which is composed of six major phyla (Firmicutes, Bacteroidetes, Proteobacteria, Actinobacteria, Spirochaetes and Fusobacteria), and 6% contains other phyla (Zhang et al., 2018). Accumulating data support a role for oral bacterial infection in the migration of various types of cells, mainly epithelial cells, and cancer cells. The effect may vary depending on the infection method, time, and cell type (**Table 1**).

**Table 1:** Summary of oral bacteria affecting cell migration.

Bacteria species	Cell types	Infection conditions	Effect on cell migration	Ref
*P. gingivalis* ATCC 33277	human immortalized oral epithelial cells (HIOECs)	MOI=1, 24 h repeatedly in 5, 10, 15, and 23 weeks	promote	(Geng et al., 2017)
	primary human oral epithelial cells (OECs)	MOI=10 and 100, 120 h	promote	(Lee et al., 2017)
	primary gingival keratinocytes	MOI=10, 24 h	inhibit	(Bhattacharya et al., 2014)
	Human telomerase immortalized gingival keratinocytes (TIGK)	MOI=100,1 h, followed by 23 h in fresh medium	promote	(Fitzsimonds et al., 2021)
	TIGK	MOI=100, 24 h	promote	(Ohshima et al., 2019)
	Human immortalized oral keratinocyte cell line OKF6/hTERT-1	MOI=100, 24 h	promote	(Hoppe et al., 2019)
	human OSCC cell lines, UM-SCC-14A (floor of mouth) and HSC-3 (tongue)	MOI=10,50,100,2 h	promote	(Kamarajan et al., 2020)
	SAS and Ca9-22 cells	MOI=1, 24 h	promote	(Inaba et al., 2014)
	OSC-20 and SAS cells	MOI=100,3 h	promote	(Ha et al., 2016)
	Human esophageal cancer cell lines NE6-T, KYSE-30 and KYSE-150	MOI=10, 48 h	promote	(Liang et al., 2020; Qi et al., 2020)
secreted gingipains of *P. gingivalis* ATCC33277	human embryonic microglia clone 3 (HMC3) cell line	MOI=1,12 h	promote	(Nonaka and Nakanishi, 2020)
Outer membrane vesicles (OMVs) of *P*. *gingivalis* ATCC 33277	OSCC cell line (HSC-3)	6 h	promote	(Liu et al., 2021b)
Ltp1 of *P*. *gingivalis* ATCC 33277	TIGK	MOI=100, 6 h	promote	(Liu et al., 2021a)
Heat-killed *P. gingivalis* ATCC 33277	OSCC cell line(H400)	MOI=100, 8 d	promote	(Abdulkareem et al., 2018a)
*P. gingivalis* W83	human buccal epithelial cell line HO-1-N-1	MOI=10,100, 17 h	inhibit	(Laheij et al., 2013)
heat-killed *P. gingivalis* W83	human buccal epithelial cell line HO-1-N-1	MOI=100, 1000,17 h	inhibit	(Laheij et al., 2013)
conditioned medium from *P. gingivalis* W83	human buccal epithelial cell line HO-1-N-1	MOI=100,17 h	inhibit	(Laheij et al., 2013)
*P. gingivalis strain 381*	Ca9-22 OSCC cells	MOI=100, 2 h	promote	(Ha et al., 2015)
*F. nucleatum* ATCC 25586	primary gingival keratinocytes	MOI=10:1, 24 h	inhibit	(Bhattacharya et al., 2014)
	human OSCC cell lines, UM-SCC-14A (floor of mouth) and HSC-3 (tongue)	MOI=10,50,100,2 h	promote	(Kamarajan et al., 2020)
	Colorectal cancer cell lines (HCT-116, LoVo)	MOI=100, 12 h and 24 h	promote	(Chen et al., 2020; Xu et al., 2021a)
Heat-killed *F. nucleatum* ATCC 10953	OSCC cell line(H400)	MOI=100, 8 d	promote	(Abdulkareem et al., 2018a)
	OSCC cell line(HOC621 cells)	MOI=10, 3 h	promote	(Shao et al., 2021)
Heat-killed *F. nucleatum* JCM8532	OSCC cell line(HOC621 cells)	MOI=10, 3 h	promote	(Shao et al., 2021)
Live or heat-killed *F. nucleatum* JCM11024	OSCC cell line(HOC621 cells)	MOI=10, 3 h	promote	(Shao et al., 2021)
Live or heat-killed *F. nucleatum* ATCC23726	OSCC cell line(HOC621 cells)	MOI=10, 3 h	promote	(Shao et al., 2021)
*T*. *denticola* ATCC35405	human OSCC cell lines, UM-SCC-14A (floor of mouth) and HSC-3 (tongue)	MOI=10,50,100,2 h	promote	(Kamarajan et al., 2020)
*P. intermedia* ATCC 25611 *T. forsythia* ATCC 43037 Conditioned medium from *Streptococcus mitis* LMG 14557 (MOI 100, 1000) and heat-killed *Streptococcus mitis* LMG 14557 Conditioned medium from *Prevotella nigrescens* ATCC 33563((MOI=100,1000) heat-killed *Prevotella nigrescens* ATCC 33563 (MOI 10, 100, 1000) Heat-killed *Prevotella intermedia* ATCC 25611 (MOI 100, 1000) *Tannerella forsythia* ATCC 43037 (MOI 50, 500)	human buccal epithelial cell line HO-1-N-1	MOI=10,100 or 1000, 17 h	inhibit	(Laheij et al., 2013)

Periodontal pathogens Porphyromonas gingivalis and Fusobacterium nucleatum promote tumor progression in an oral-specific chemical carcinogenesis model.

*P. gingivalis*, the key etiological agent in periodontitis, can successfully invade oral epithelial cells and live intracellularly. It adheres and internalizes to oral cells by modulating host signaling cascades such as phosphorylation/dephosphorylation of molecules and changes in the cell cytoskeleton (Andrian et al., 2006). For instance, *P. gingivalis* can induce gingival epithelial cells to undergo autophagy and traffic into autophagosome vacuoles to protect itself from targeted lysosome degradation mediated by selective ubiquitin (Lee et al., 2018). *P. gingivalis* expresses virulence and releases outer membrane vesicles (OMVs), which are involved in the communication between bacteria and the host. In general, *P. gingivalis*, its derivatives (e.g., heated-killed or conditioned medium from bacteria) and secreted substances (OMVs and Ltp1) can promote the migration of oral epithelial cells and OSCC cells (Inaba et al., 2014; Ha et al., 2015; Ha et al., 2016; Geng et al., 2017; Lee et al., 2017; Abdulkareem et al., 2018a; Hoppe et al., 2019; Ohshima et al., 2019; Kamarajan et al., 2020; Fitzsimonds et al., 2021; Liu et al., 2021a; Liu et al., 2021b). Comprehensive analysis of the host transcription response to *P. gingivalis* infection showed the mode of increased cell migration (Geng et al., 2019). The colonization of *P. gingivalis* has been identified as a risk factor for OSCC and is related to prognosis (Ganly et al., 2019). However, the inhibitory effect has also been observed in some studies (Laheij et al., 2013; Bhattacharya et al., 2014). This may suggest that bacterial challenge may hinder the re-epithelialization process and lead to delayed healing after the barrier function of the gingival epithelium is destroyed. We speculated that the possible reason for such a diametrically opposed situation is the relatively short infection time or different sources and locations of cells. It may also be due to differences in protocols between continuous infection and long-term culture with a short infection followed by a medium change. When serial infection experiments with *P. gingivalis* are performed *in vitro*, cells often float alive due to strong protease activity.

As a symbiotic bacterium, *F. nucleatum* is commonly found in the oral cavity. It expresses a variety of adhesins, such as FadA, on the surface to adhere to and invade host cells. Similarly, *F. nucleatum* can also release extracellular vesicles (EVs) or OMVs (Liu et al., 2019; Liu et al., 2021). Studies have shown that infection with *F. nucleatum* ATCC 25586 and heated-killed *F. nucleatum* ATCC 10953 promotes OSCC cell migration and invasion *in vitro* (Abdulkareem et al., 2018a; Kamarajan et al., 2020; Shao et al., 2021). The effect of its derivatives, secretions and virulence factors on cell migration still needs further study.

Butyric acid (BA), a short-chain fatty acid, is produced by periodontopathic bacteria, such as *P. gingivalis* and *F. nucleatum.* Large amounts of BA have been detected in the oral cavities of patients with periodontal disease. BA from periodontopathic bacteria does not directly affect the migration of ameloblastomas but has an indirect influence through the expression of epidermal growth factor (EGF) and transforming growth factor β1 (TGF-β1) (Ishikawa et al., 2020). Other oral bacteria are also related to cell migration. Oral streptococci, such as *Streptococcus gordonii*, is an important component of the oral microbiome. *S. gordonii* has been considered an early colonizer to host tissues. It can resist ZEB2 induction by *P. gingivalis* and then inhibit cell migration (Ohshima et al., 2019). The specific molecular mechanism of oral bacteria related to cell migration, interaction between different kinds of bacteria and its effect on cell migration deserve further study.

There is already evidence that oral bacteria can spread to other body sites through the bloodstream or digestive tract and participate in a variety of systemic diseases (Fiorillo et al., 2019). It may affect the pathogenesis and progression of diseases *via* cell migration and metastasis. *P. gingivalis* increases the migration of esophageal squamous cell carcinoma (Liang et al., 2020; Qi et al., 2020). *F. nucleatum* has been found to be enriched in colorectal cancer (CRC) tissue and acts as a pro-carcinogenic bacterium (Wang et al., 2021). It promotes CRC cancer migration *in vitro* and metastasis *in vivo* (Chen et al., 2020; Xu et al., 2021a). In addition, the metastases in mice in which breast cancer developed after infection with *F. nucleatum* were larger than those in the control group (Parhi et al., 2020). *P. gingivalis* has been found in the brains of Alzheimer’s disease patients and is involved in the disease process (Dominy et al., 2019). Gingipains, cysteine proteases secreted by *P. gingivalis*, induces cell migration and membrane ruffling in the human embryonic microglia clone 3 (HMC3) cell line through the protease-activated receptor 2 (PAR2)/ERK1/2 pathway (Nonaka and Nakanishi, 2020). This signaling pathway may be necessary for embryonic microglial cells to move into the infection sites.

### Emerging Link Between Oral Virus/Fungi and Cell Migration

Dental professionals are usually familiar with viruses present or cause symptoms in the oral cavity. In a broad sense, they can both be defined as oral viruses. There is not always a clear distinction between them. For example, viruses that induce symptoms affecting oral tissues can be present in the oral cavity following replication and release from other tissues or the blood circulation. Prolonged infection of certain viral genes can insert into host DNA as proto-oncogenes to turn them into oncogenes, leading to malignant transformation (Young et al., 2016; Warburton et al., 2018). There is emerging evidence that Epstein–Barr virus (EBV), as an oncogenic virus, promotes OSCC progression. P53 can promote the emergence of leader cells and then coordinate the migration of epithelial cells (Kozyrska et al., 2022). EBV decreases the stability of P53 and increases the expression of matrix metalloproteinase (MMP) through CTAR family proteins/programmed cell death protein 1 ligand, thereby promoting OSCC cell metastasis and tumorigenesis (Radaic and Kapila, 2021). Human papillomavirus (HPV) is detectable in 40-70% of oropharyngeal cancer (OPC) and 20% of non-OPC, almost the HPV16 subtype (Mehanna et al., 2013). The relationship between HPV and cervical cancer has been generally acknowledged. HPV16 can lead to enhanced migration and invasion of cervical cancer cells *in vitro* and in a mouse model (Hu et al., 2015; Wang et al., 2019).

Fungi are common, albeit minor, members of the oral microbiota, which are structurally and metabolically distinct from other oral microorganisms, such as bacteria and viruses. They have varied cell morphologies, including yeast, hyphae, pseudo hyphae, and chlamydospores. Although filamentous fungi can rarely be isolated from the oral cavity, yeast can be cultured from the saliva of approximately 40% of individuals, such as *Candida tropicalis*, *Candida dubliniensis*, and *Candida glabrata*. The most frequent oral fungal infection is *Candida albicans*. *C. albicans* significantly increased in OSCC patients compared with the control group (Hong et al., 2020). *C. albicans* can enhance the migratory ability of oral keratinocytes through activation of the ERK/focal adhesion kinase (FAK) pathway (Shi et al., 2009). Exposure to different concentrations of *C. glabrata and Candida kefyr* (MOI=1,10) was found to inhibit human buccal epithelial cell migration *in vitro* (Haverman et al., 2017). This inhibitory effect may play a role in the process of ulcerative mucositis.

## The Mechanisms of Oral Microbiota Regulating Cell Migration

**Figure 1:**
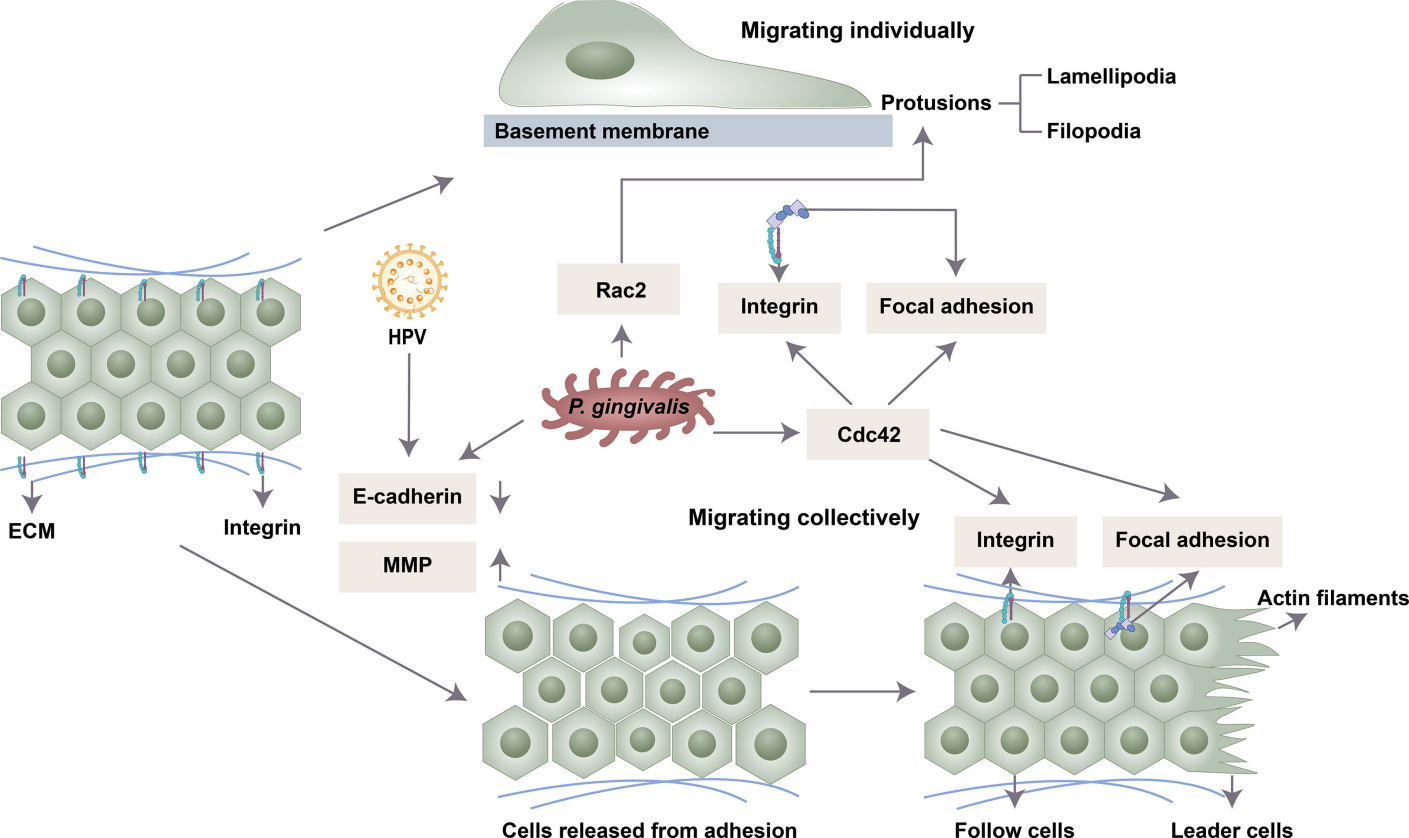
Possible mechanisms by which oral microbiota influence cell migration. *P. gingivalis* and HPV 16 release intercellular adhesions by downregulating E-cadherin and upregulating MMP. *P. gingivalis* enhances the expression of Cdc42 and Rac2 to induce the front and back polarity that is crucial for cell migration. *P. gingivalis* drives cell clusters to move orientally through the formation of integrin and focal adhesion.

Migration is a multistep process. First, the cells are polarized in response to the migration-promoting stimulus, and then the membrane protrusions extend in the direction of the stimulus (Lauffenburger and Horwitz, 1996). Then, the adhesion of the protrusions to the ECM produces traction, which allows the movement of the cell, disassembly of the adhesion points and retraction of the back of the cell (Ridley et al., 2003). Collective and single-cell migration share these broad mechanistic characteristics (Yang et al., 2019). Studies have found that oral microbes mainly regulate cell migration by affecting cell adhesion, polarization, guidance, etc. (**Figure 1**).

### Adhesion

Intercellular adhesion is mediated by different types of junction complexes, including tight junctions, adhesive junctions, gap junctions and desmosomes (Rusu and Georgiou, 2020). Oral microbiota can degrade epithelial cell tight junction proteins in an MMP-dependent manner or by affecting the activity of transcription factors (Ha et al., 2015). The shape of infected cells became slender, indicating absent contact inhibition, and cell junctions were weakened under a transmission electron microscope (Geng et al., 2017). MMP is involved in focal degradation of type IV collagen, breakdown of basement membrane and release of cell adhesion (Strutz et al., 2002). MMP-1, MMP-2, MMP-3, MMP-7 and MMP-9 were upregulated following exposure to *P. gingivalis* stimulation (Ha et al., 2016; Lee et al., 2017). One mechanism is that gingipains of *P. gingivalis* process the proenzyme of MMP-9 into active MMP-9 to promote cell migration and invasion (Inaba et al., 2014; Inaba et al., 2015). This process is regulated by the PAR4, p38/HSP27, ERK1/2-Ets1, and PAR2/NF-κB pathways (Inaba et al., 2014; Inaba et al., 2015). *P. gingivalis* could also activate the expression of MMP-2 and MMP-9 by increasing the transcription of nicotinamide N-methyltransferase (NNMT) and Gas6 (Tang et al., 2011; Geng et al., 2017).

E-cadherin, the central protein of cell–cell adherens junctions, is downregulated by a series of transcription factors (Slug, Snail, JAG1, Notch, Zeb1 and Zeb2) (Wu and Zhou, 2010). *P. gingivalis* can significantly enhance the activity of these transcription factors (Geng et al., 2017). Moreover, *P. gingivalis* infection could inhibit the expression of E-cadherin and increase the expression of Snail in oral epithelial cells by upregulating the transcription of colon cancer associated transcript 1 (CCAT1) and growth arrest specific 6 (Gas6) (Jiang et al., 2015; Guo and Hua, 2017). HPV-16 may also facilitate the migration and metastasis potential of cervical cancer through altered cadherin switching (Hu et al., 2015).

### Polarization and Guidance

Polarity is characteristic of epithelial cells. In polarized epithelial cells, the intercellular junction complex is located asymmetrically. The spatial asymmetry of these complexes is mediated by a class of evolutionarily conserved proteins, which can be divided into three functional groups: the Crumbs2 complex, Scribble complex and Par complex. The apical domain is related to the Crumbs complex, the basolateral domain is composed of the Scribble complex, and the subapical region of the apical-basal boundary is related to the Par complex, which is composed of Par3, Par6, and atypical protein kinase C and Cdc42.

In single-cell migration, migrating epithelial cells tend to lose apical-basal polarity and rearrange their actin cytoskeleton (Gibieža and Petrikaitė, 2021). The protusions of the leading edge drive the cell to orientally migrate, and the main force of migrating is generated by the lamellipodia. Myoslin II and Rho catalyze the formation of new cell-ECM adhesion (focal adhesions) during lamellipodia extension. The stress fibers are joined to mature focal adhesions, connecting the cell to the ECM. In collective migration, cells in different positions show different expression patterns. The simple case is front/rear polarization. The leading cells with a mesenchymal phenotype guide the following cells to retain epithelial characteristics. This process is coordinated by cell-ECM and cell–cell interactions.

Rho family GTPases include RhoA, Rac and Cdc42 proteins, which participate in the formation of cytoskeletal components by inducing the accumulation of F-actin in the front of the cell (Ridley, 1999). Rho GTPases regulate cytoskeletal actin rearrangement and thus cell dynamics (Sahai and Marshall, 2003; Lawson and Ridley, 2018). Polar proteins regulated by GTPase enzymes of the Rho family induce the formation of the front and rear axes (Capuana et al., 2020). Rac protein is closely related to the formation of lamellipodia and cell migration. The Rho-dependent localization of myosin IIB in the back of the cell is necessary for the maintenance of front and back polarity and tail contraction during the process of mesenchymal migration (Vicente-Manzanares et al., 2008). Cdc42 mainly produces front and back polarity. The local activation of Cdc42 and its spatial gradient in nonpolarized cells drive the formation of the initial protruding front under uniform chemotaxis stimulation (Yang et al., 2016). When the apical junction complex is destroyed, Cdc42 and the polar protein complex relocate from the tight junction area to the front and induce the centrosome and Golgi to relocate to the front of the cell, promoting the growth of microtubules to the front of the cell and subsequent cell migration (Burute et al., 2017). Therefore, at the front end, Cdc42 and Rac promote actin polymerization, thereby promoting the formation of protrusions such as filopodia or lamelia. Increased actin reorganization and Cdc42 and Rac activity are observed in OSCC (Iwai et al., 2010). *P. gingivalis* enhanced the expression of Rac2 and Cdc42 in platelets and neutrophils (Börgeson et al., 2011; Senini et al., 2019). *P. gingivalis* fimbriae induced transendothelial migration of monocytes by activating Rac1 and PI3K (Harokopakis et al., 2006). Its effect on oral epithelial cells needs further study.

Integrin alpha V and FAK signals help mediate cell migration (Trepat et al., 2012).Cell migration depends on the binding of integrins to the ECM, which activates downstream signaling, including FAK phosphorylation and mitogen-activated protein kinase (MAPK), to recruit focal contacts. Several integrins, such as beta-6 integrin, can facilitate cell migration through actin cytoskeletal reorganization and cell polarization. *P. gingivalis* inhibits the induction of integrin beta-3 and -6 and the cell migratory process in oral keratinocytes (Bhattacharya et al., 2014). Periodontal pathogens (*P. gingivalis, F. nucleatum*, and *T*. *denticola*) promote OSCC cell migration through the activation of integrin alpha V and FAK (Kamarajan et al., 2020). The E6 protein of HPV could also promote actin cytoskeleton assembly through β1-integrin signaling (Holloway and Storey, 2014).

### EMT

**Figure 2:**
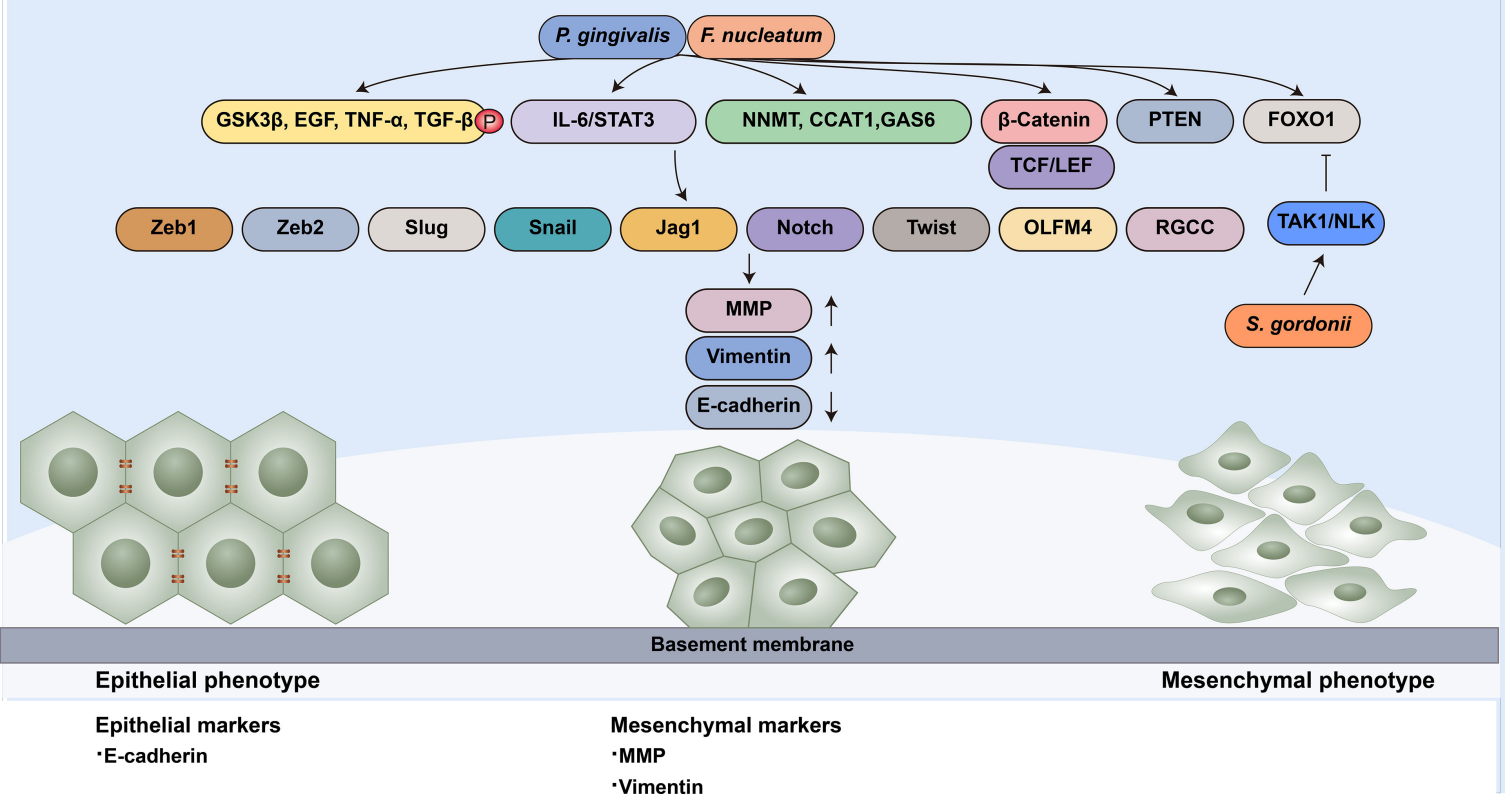
Oral microbiota guide epithelial-mesenchymal transition of normal epithelial cells. EMT-associated transcription factors (Zeb1, Zeb2, Slug, Snail, Jag1, Notch, Twist, OLFM4 and RGCC) can induce epithelial cells to undergo partial or complete mesenchymal transformation by downregulating epithelial markers (such as E-cadherin) and upregulating mesenchymal markers (MMP and Vimentin). *P. gingivalis* and *F. nucleatum* enhance the expression of EMT-associated transcription factors through diverse pathways: (1) the phosphorylation of GSK‐3β, EGF, TNF-α and TGF-β1; (2) the activation of the IL-6/STAT3 pathway; (3) the upregulation of NNMT, CCAT1 and GAS6;(4) the nuclear translocation of β-catenin and further activation TCF/LEF promoter elements; (5) the activation of PTEN; and (6) the upregulation of FOXO1 *S. gordonii* can resist EMT-associated factor induction by *P. gingivalis* by suppressing FOXO1 and activating the TAK1-NLK negative regulatory pathway.

The other strategy employed by oral microbiota to manipulate cell migration is modulating the EMT process (**Figure 2**). Specifically, microbial dysbiosis results in the degradation of epithelial tight junction proteins, enhances mesenchymal characteristics, and induces at least a portion of the EMT process. For instance, *P. gingivalis* regulates epithelial barrier function through the degradation of E-cadherin (Sztukowska et al., 2016; Abdulkareem et al., 2018a). *P. gingivalis* and *F. nucleatum* could also enhance the expression of EMT-associated transcription factors (Zeb1, Zeb2, Slug, Snail, Jag1, Notch, Twist, OLFM4 and RGCC) in oral epithelial cells and OSCC cells through diverse pathways, including the phosphorylation of glycogen synthase kinase-3β (GSK‐3β), EGF, tumor necrosis factor-α (TNF-α) and TGF-β1 (Bhattacharya et al., 2014; Sztukowska et al., 2016; Abdulkareem et al., 2018a; Abdulkareem et al., 2018b). *S. gordonii* can resist ZEB2 induction by *P. gingivalis* by suppressing FOXO1 and activating the TAK1-NLK negative regulatory pathway (Ohshima et al., 2019). *F. nucleatum* can activate the signal transducer and activator of transcription 3 (STAT3) signaling pathway, which can increase the expression of EMT-associated genes (E-cadherin, Snail and Twist) (Huang et al., 2011; Wang et al., 2020). The upregulation of partial EMT genes is also observed in *F. nucleatum*-infected OSCC cells (Shao et al., 2021).

Wnt/β-catenin facilitates EMT in cancer progression (Liu et al., 2017; Li et al., 2021). β-catenin in the nucleus binds to the T cell factor/lymphoid enhancer factor (TCF/LEF) transcription factor, the major end point mediators of Wnt/β-catenin signaling, to activate EMT. Vimentin is the target of Wnt/β-catenin signaling. The nuclear translocation of β-catenin and activation of vimentin can be observed in OSCC cells, which are related to poor prognosis (Chaw et al., 2012). The gingipains of *P. gingivalis* modulate the β-catenin pathway in gingival epithelial cells and the disassociation of the β-catenin destruction complex composed of scaffolding proteins and the kinases GSK3β and Casein Kinase 1α (CK1α). It can induce nuclear translocation of β-catenin and further activate TCF/LEF promoter elements (Zhou et al., 2015).

It has been proven that several genes are positively relevant to cell migratory and invasive ability, such as CCAT1, NNMT and Gas6. CCAT1 and Gas6 can downregulate E-cadherin and upregulate EMT-associated transcription factors, such as Snail and Twist (Jiang et al., 2015; Guo and Hua, 2017). The mRNA expression of NNMT, CCAT1 and GAS6 was increased in *P. gingivalis*-infected oral epithelial cells (Geng et al., 2017).

### Others

Keratin 7 (KRT7) is a type II cytokeratin and is involved in cell motile activity. It has been proven to be relevant to lymph node metastasis and poor prognosis in CRC (Bayrak et al., 2011; Hrudka et al., 2021). *F. nucleatum* upregulates the long noncoding RNA KRT7-antisense and stabilizes KRT7 mRNA *via* the NF-κB pathway (Chen et al., 2020). HPV-16 enhances actin polymerization by downregulating alpha-actinin-4 (ACTN4), leading to enhanced migration and invasion (Tentler et al., 2019; Wang et al., 2019).

## Critical Techniques for Investigating Cell Migration

With the development of new microscopy methods and fluorescent reagents specifically used for cell imaging, microscopy technology plays a central role in the research of cell biophysics. Optical microscopes allow us to view cell structures with previously unattainable spatial and temporal resolution and to image living cells in tissues and animals. Improvements in electron microscopy technology allow us to understand the molecular structure of organelles in cells in more detail. In recent years, an increasing number of technologies have emerged, such as video real-time microscopy, confocal microscopy, multiphoton microscopy, intravital microscopy, superresolution fluorescence microscopy, electrochemiluminescence microscopy, and traction force microscopy, which make the observation and analysis of cell migration more accurate and intuitive (Horwitz, 2016).

Most biological research relies on conventional experimental techniques, and static analysis is only allowed at certain time points *in vitro*. Visualizing cell dynamics in organisms can provide opportunities to study key biological phenomena *in vivo*. However, electron microscopy is usually not suitable for live or wet samples due to the need for vacuum operation conditions. Soon after the first compound microscope was invented in 1595, the intravital microscope (IVM) was used for physiological research. IVM can be combined with a variety of optical systems, such as confocal and multiphoton microscopy, to conduct deeper observations of tissues and directly observe the biological structure and dynamic behavior of objects, including single cells and living animals. With these characteristics, IVM can be used to visualize the biological morphology of various fields, such as vascular biology, immunology, stem cell biology and oncology (Choo et al., 2020). For instance, in the field of oncology, IVM can be used to observe the single-cell behavior of cancer cells and immune cells during tumor progression and metastasis (Ng et al., 2008; Pinner et al., 2009; Gabriel et al., 2018; Kuo et al., 2019). To overcome the obstacle of the traditional IVM that the limited frame rate cannot accurately observe the rapid dynamic behavior of cells, a real-time IVM capable of video image scanning (over 30 frames/sec) has been invented to visualize faster cell movement and further study cell functions and the interactions between cells (Padera et al., 2002). Moreover, the Boyden chamber assay and scratch wound assay are standard techniques for studying cell invasion and migration, but both techniques have their own limitations. The Boyden chamber assay is difficult and time-consuming, while the scratch wound assay has low repeatability. The real-time video microscope can be introduced into the incubator to generate real-time images of cell migration, which can provide accurate quantitative data for wound healing and is used to provide automatic real-time analysis of cell migration, which improves repeatability (Jain et al., 2012). This video microscope-based scratch experiment has proven to be a reliable technique for evaluating cell migration and invasion (Guy et al., 2017).

In addition, superresolution fluorescence microscopy, harmonic generation microscopy, electrochemiluminescence microscopy, and traction force microscopy are all powerful tools for studying cell migration. The5 superresolution microscope overcomes the limitations of conventional optical microscopes in resolution, dimensionality, quantification, and imaging speed. It can be used to observe the cellular processes of single cells with nanometer-level resolution, contributing to the understanding of rapid cell dynamics with a more visualized subcellular and molecular scale. Increasing methods have been developed to achieve superresolution, such as stimulated emission depletion (STED), structured illumination microscopy (SIM), photoactivation localization microscopy (PALM), stochastic optical reconstruction microscopy (STORM), and superresolution optical fluctuation imaging (SOFI) (Feng et al., 2018). These realize the nanoscale, visualization and quantitative analysis of signaling pathway molecules such as membrane proteins (Xu et al., 2021b). Second harmonic generation (SHG) is a second-order nonlinear optical process. Two photons interacting with nonlinear optical materials, such as collagen, combine to form a new photon whose frequency is twice that of the original photon, and the wavelength is halved (Wolf et al., 2009). SHG microscopy is a high-resolution nondestructive imaging method that represents an ideal method for detecting the geometry of collagen in natural and/or connective tissues. It has been used to study the morphology of fibrous collagen in a variety of tissues, which helps to understand the structure of collagen in tissues under normal or abnormal conditions. Unlike SHG microscopy, which requires specific asymmetry of the imaging structure, third harmonic generation (THG) is a combination of three photons converted into a photon with one third of the excitation wavelength and three times the energy. Therefore, compared to SHG, the application range of THG is wider. The combination of SHG or THG with fluorescence detection and intravital microscopy provides more details about tissue-tissue and cell-tissue interactions, which helps to simulate the migration of tumor cells in the tissue in a specific environment (Weigelin et al., 2012). Electrochemiluminescence microscopy can provide a clear visual contrast between the adhesion site and the noncontact domain so that the former can be selected and displayed in a label-free manner to image the cell matrix adhesion of the moving cell clusters to study the movement of cells in collective migration (Ding et al., 2020). Traction force microscopy is an experimental technique used to quantify the contractile force produced by adherent cells by placing the cells on a flexible material, such as polyethylene glycol or polyacrylamide gel with a known elastic modulus, track the displacement of the substrate caused by the cell contraction, and then convert it into a traction field (Hur et al., 2020). Traction force microscopy with integrated microfluidics can precisely control physical and chemical stimulation to detect the migration speed, traction and intercellular tension of cell clusters under different chemical gradients (Jang et al., 2019).

In the limited three-dimensional (3D) space, the situation of cell migration is more complex, which is difficult to observe by traditional methods. New engineering model systems, such as hydrogels and microchannel assays provide new insights into 3D cell migration. The synthesized hydrogel can independently modulate the hardness, composition, degradability, or other characteristics to analyze the role of a single characteristic of ECM in cell migration (Trappmann et al., 2017). Therefore, it provides a platform to see how cancer cells respond to different biochemical and mechanical signals. Other types of cells can also be introduced into these hydrogels to simultaneously observe cell-matrix and cell-cell interactions (Katz and West, 2022). Confined microchannel approach can introduce and adjust the interface geometry to explore changes in cytoskeleton, adhesion, and regulatory proteins induced by different experimental microenvironments (Mak et al., 2011). Polydimethylsiloxane (PDMS) microchannel devices are also widely used to study migration in 3D confinement. Microchannel system allows direct and real-time imaging and explores the mechanism of migration under confined conditions without shear stress. Microchannel coated with different ECM proteins can be applied to explore cells’ response to external gradients. In addition, the limited migration space *in vivo* can also be simulated by grooved substrates, micropatterned lines and islands, vertical confinement, patterned gels, and so on (Paul et al., 2016).

## Summary

Studies have shown that the oral microbiota can regulate the carcinogenic process of cells, and this process may involve abnormal cell migration. Partial or complete EMT is related to increased migratory ability and participates in cancer invasion and metastasis. Specific genetic changes and signaling pathway activation during cell migration may also be seen in EMT, tumorigenic phenotypes and cancer metastasis. Collective migration is the main form of cancer invasion, and it is associated with worse prognosis. Oral microbial dysbiosis is related to abnormal cell migration of oral epithelial cells. Increasing evidence has shown that *P. gingivalis*, *F. nucleatum*, *Streptococci* and their virulence factors regulate cell migration, whose effects vary with infection methods, time, and cell types. Some oral viruses and fungi can affect cell migration, and further study is needed. The oral microbiota also participates in systemic diseases through cell migration. The mechanism by which the oral microbiota regulates cell migration includes cell adhesion, polarization, guidance, EMT, etc. real-time microscopy, superresolution microscopy, harmonic microscopy and traction microscopy are widely used to observe and analyze the migration process. We expect that with further development of microscope imaging technology, the relevant mechanism can be observed more intuitively and accurately.

## Author Contributions

HB and JY drafted the manuscript. SM and CL edited and added valuable insights to the manuscript. All authors approved the final manuscript and agreed to be accountable for all aspects of the work.

## Funding

This study was supported by the National Natural Science Foundation of China (82071108).

## Conflict of Interest

The authors declare that the research was conducted in the absence of any commercial or financial relationships that could be construed as a potential conflict of interest.

## Publisher’s Note

All claims expressed in this article are solely those of the authors and do not necessarily represent those of their affiliated organizations, or those of the publisher, the editors and the reviewers. Any product that may be evaluated in this article, or claim that may be made by its manufacturer, is not guaranteed or endorsed by the publisher.

## Abbreviations

*Porphyromonas gingivalis*, *P. gingivalis*; *Fusobacterium nucleatum*, *F. nucleatum*; *Treponema denticola*, *Treponema denticola*; epithelial to mesenchymal transition, EMT; Actin-binding proteins, ABPs; discoidin domain receptor 1, DDR1; oral squamous cell carcinoma, OSCC; extracellular matrix, ECM; outer membrane vesicles, OMVs; extracellular vesicles, EVs; Butyric acid, BA; epidermal growth factor, EGF; transforming growth factor β1, TGFβ1; *Streptococcus gordonii*, *S. gordonii*; Epstein–Barr virus, EBV; matrix metalloproteinase, MMP; human papillomavirus, HPV; oropharyngeal cancer, OPC; nicotinamide N-methyltransferase, NNMT; growth arrest specific 6, Gas6; focal adhesion kinase, FAK; T cell factor/lymphoid enhancer factor, TCF/LEF; glycogen synthase kinase-3β, GSK‐3β; tumour necrosis factor-α, TNF-α; Keratin 7, KRT7; intravital microscope, IVM; Second harmonic generation, SHG; third harmonic generation, THG; three-dimensional, 3D; polydimethylsiloxane, PDMS.
